# A comparison of manual therapy and active rehabilitation in the treatment of non specific low back pain with particular reference to a patient's Linton & Hallden psychological screening score: a pilot study

**DOI:** 10.1186/1471-2474-8-106

**Published:** 2007-11-01

**Authors:** Elaine Hough, Richard Stephenson, Louise Swift

**Affiliations:** 1 Physiotherapy Department, St Helen's Hospital, Marshalls Cross Road, St Helens, Merseyside, UK; 2 School of Allied Health Professions, University of East Anglia, Norwich, UK; 3 School of Medicine, Health Policy and Practice, University of East Anglia, UK

## Abstract

**Background:**

Clinical guidelines for the management of back pain frequently recommend 'manual therapy' as a first line intervention, with psychosocial screening and 'active rehabilitation' for those not improving at 6 weeks post onset. The potential for psychosocial factors to predict treatment response and therefore outcome has not been adequately explored. The purpose of this pilot study was to determine the feasibility of a study to compare manual therapy and active rehabilitation outcomes for subjects with sub-acute/chronic back pain, investigate whether any difference in outcome was related to psychosocial factors, and to inform the design of a main study.

**Methods:**

A convenience sample of 39 patients with non-specific low back pain referred to the physiotherapy department of an acute NHS Trust hospital was recruited over a nine month period. Patients completed the Linton and Hallden psychological screening questionnaire (LH) and were allocated to a low LH (105 or below) or high LH (106 or above) scoring group. The low or high LH score was used to sequentially allocate patients to one of two treatment groups – Manual Therapy comprising physiotherapy based on manual means as chosen by the treating therapist or Active Rehabilitation comprising a progressive exercise and education programme – with the first low LH scoring patient being allocated to active rehabilitation and the next to manual therapy and so on. Treatment was administered for eight sessions over a four-week period and outcome measures were taken at baseline and at four weeks. Measures used were the Roland Morris Questionnaire (RMQ), two components of the Short Form McGill (total pain rating index [PRI] and pain intensity via visual analogue scale [VAS]), and the LH.

**Results:**

The manual therapy group demonstrated a greater treatment effect compared with active rehabilitation for RMQ (mean difference 3.6, 95% CI 1.1 – 6.2, p = 0.006) and PRI (7.1, 95% CI 2.0 – 12.2, p = 0.007) and marginally significant results for VAS (15, 95% CI -1.1 to 31.2, p = 0.067). A linear model allowing for confounding effects and the interaction between high or low LH scores supported these results. The interaction effect was not significant for any outcome measure but this could be due to an insufficient number of subjects to detect this effect.

**Conclusion:**

Comparative evaluation of manual therapy and active rehabilitation with reference to LH psychosocial scores is likely to be detectable by the methods used here. However several alterations to the study design are recommended for the main study. A pragmatic trial using a randomisation process with stratification on the LH score and priori power analysis to determine sample size are suggested for the main study.

## Background

Non specific low back pain is one of the most common causes of disability affecting approximately 17.3 million people in the United Kingdom with direct costs to the National Health Service (NHS) of £1 billion per annum [1]. Current clinical guidelines for the management of non specific low back pain vary but generally recommend a primary intervention of manual therapy, during the acute stage, with an active rehabilitation programme for those patients not recovering beyond 6–12 weeks duration [2,3]. However, despite widespread acceptance, such guidelines tend to be consensus rather than evidence based with limited, and conflicting, evidence to support the use of manipulation [4-6] or exercise [7-9].

Several recent randomised control trials have compared physiotherapy and/or manual therapy with other management approaches for non specific low back pain but there remains conflicting and insufficient evidence from which strong conclusions may be drawn. Trials have variously suggested that: physiotherapy may be no more effective than one advice session [10]; spinal stabilisation may be more effective than manual therapy [11]; spinal stabilisation and manual therapy is no more effective than exercises and stabilisations, but both treatment approaches do show improvement over baseline [12]; manual therapy may be more effective than active rehabilitation in reducing pain, disability and improving general health and return to work [13]; manual therapy is better than stay active campaigns according to pain and disability rating[14]; manual therapy with specific exercises is more effective than manual therapy with no-specific exercise [15]. In the largest pragmatic randomised trial in the United Kingdom (UK BEAM Trial), 1334 patients were allocated to one of four main treatment arms with spinal manipulation and exercises showing greater improvements (over spinal manipulation alone, exercise alone and General Practitioner 'best practice') at 3 and 12 months based primarily on the Roland Morris Disability Questionnaire and Euroquol 5D, although the relative increases in function were small [16,17]. These findings were supported by patient perceptions of treatment, where general practitioners were perceived as non-experts and manual therapists perceived as hands-on experts, with a stronger perceived benefit from non-passive therapies [18] that may positively influence the findings. The trial also recommended an economic cost-effectiveness in favour of manipulation and exercise or manipulation alone [19].

There is increasing hypothecation that current practice and outcome measures are based on an assumed structural relationship between the cause and perception of non-specific low back pain [2] leading to some studies attempting to profile subjects towards treatment approaches [20-23]. Where psychosocial factors are known to influence pain perception [24] the analysis of such factors may identify patients who have a higher risk of developing long term disability [25]. Several researchers have specifically explored the effects of intervention on psychosocial status, or the influence of psychosocial factors on treatment outcomes [26-30]. Wand et al, [31] found that early intervention (compared to leave alone) had greater improvements in terms of disability, mood, general heath and quality of life at six weeks, and whilst disability and pain showed no greater difference in improvement between groups at six months, mood, general health and quality of life remained significantly improved.

### Aims

We decided to conduct an exploratory pilot study with two specific aims:

1. To inform the design and feasibility of a future study of this subject.

2. To *provisionally *compare the effectiveness of manual therapy and active rehabilitation as first line interventions for patients referred to physiotherapy with non-specific low back pain of duration longer than 6 weeks, and whether this effect differs according to psychosocial status. Specifically to:

• Compare the effect of a manual therapy or active rehabilitation treatment intervention. The Roland Morris (RMQ) functional status questionnaire, the Pain Rating Index (PRI) and Visual Analogue Scale (VAS) components of the Short Form (SF) McGill pain questionnaire were selected as outcome measures.

• Investigate whether any difference found varies according to the subjects' psychosocial status as determined by an initial Linton and Hallden (LH) questionnaire score of 106 and above (high LH) compared with below 106 (Low LH).

## Methods

**Figure 1:**
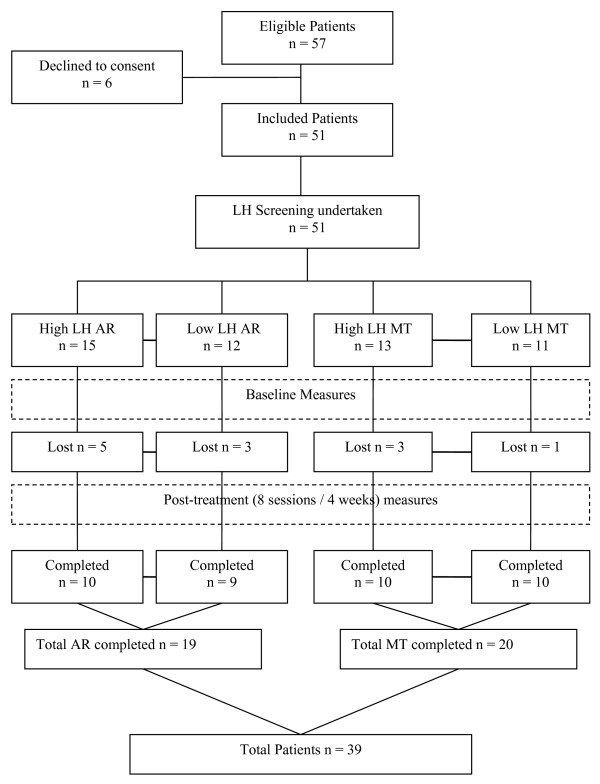
**Modified Consolidated Standard for reporting clinical trials (CONSORT) diagram for method [59]**. MT (manual therapy); AR (active rehabilitation); LH (Linton and Hallden Psychological Screening Score): low (below 106) or high (106 or above).

The recruitment and progress of patients is offered as a CONSORT (consolidated standard for reporting clinical trials) diagram [59] in Figure 1.

### The sample

A convenience sample of all patients with non-specific low back pain referred to the physiotherapy department of an acute NHS Trust hospital over a 9-month period was used. Patients who consented were included if they: were referred with somatic non-specific low back pain (with or without leg pain) with duration of 6 weeks or longer; were able to travel independently to the hospital; were literate in the 'English' language sufficient to complete the outcome questionnaires; were aged between 18 and 70 years. Patients were excluded if they had: received physiotherapy of any kind within the last 3 months; participated in regular sporting activity similar to the active rehabilitation programme; any indication of unstable neurological signs; any known underlying pathology or systemic condition that may preclude the exercises associated with active rehabilitation; undergone spinal surgery within the last year.

Four physiotherapists, who would normally accept such referrals in this specific setting, were involved with recruitment into the study. Informed consent was obtained from every participant by the recruiting physiotherapist. For those patients who did not wish to participate, management options were discussed as per normal departmental practice and a 'follow up' appointment was arranged outside the study without delay. No patient was returned to the waiting list. The researcher gave specific verbal and written instruction to each recruiting physiotherapist to standardise the information given to the patient.

The LH questionnaire was administered at the point of entry into the study and patients allocated to a high LH score group (106 or above) or a low LH score group (below 106). Patients were then allocated to manual therapy or active rehabilitation treatment groups in sequence by their LH score, such that the first 'low' LH scoring patient was allocated to active rehabilitation, the second to manual therapy, the third to active rehabilitation, the fourth manual therapy and so on. Given the small sample size and limited time frame for the study, this ensured a similar number of 'high' and 'low' LH scores in each treatment group. On recruitment to the study baseline outcome measures were collected. The recruiting physiotherapist placed all the completed questionnaires into a sealed brown envelope thus ensuring blinding of the researcher and treating physiotherapist to the baseline scores.

The outcome measure questionnaires were completed for the second time after the interventions and returned by the patient in a sealed brown envelope. To reduce bias the researcher played no part in study other than outlined and all treating physiotherapists were blind to the LH scores and client self-report scores throughout.

All subjects were given the opportunity of the alternative treatment at the end of their allocated treatment.

### The interventions

Subjects allocated to the active rehabilitation group received a progressive exercise and education programme based on that of Klaber Moffat & Frost [32], involving twice-weekly attendance over 4 weeks with programmes commencing every month. All subjects allocated to the active rehabilitation group joined the next available programme along with non-study patients. One Senior I (higher clinical grade) and one Senior II (lower clinical grade) physiotherapist delivered the active rehabilitation as part of their normal working routine throughout the period of data collection; the same physiotherapists conducted each session of all programmes which ensured some degree of standardisation across programmes. Both therapists were equally experienced in the delivery of the programme.

The manual therapy group received physiotherapy based on manual means, including related home exercises. Treatment given was based on clinically reasoned assessment findings and treatment goals, mutually agreed between therapist and subject. Physiotherapists were free to select any treatments, limiting homogeneity and standardisation, but representing 'normal' practice that is increasingly accepted in clinical research [33]. Post-hoc analysis identified the techniques used as spinal manipulation and mobilisation; mobilisation of neural structures; muscle balance techniques; specific home exercises linked to treatment techniques given. Patient education and discussion of clinical findings were offered, together with an initial prognosis. Advice to remain active and gradually return to normal activities was also given.

Two Senior II physiotherapists, who worked in the outpatient department, treating patients with non-specific low back pain as part of their normal working routine, were responsible for manual therapy treatment intervention throughout the period of data collection. If improvement to discharge, occurred in the manual therapy group before the eight treatments were completed treatment was stopped accordingly, but a review appointment was made for the fourth week for the client to complete and return the outcome questionnaires. In such circumstances the treating physiotherapists were advised to make specific note and highlight this on the front of the patient's card. Where further treatment beyond four weeks was deemed appropriate in the manual therapy group, final outcome questionnaires were completed at four-weeks and treatment continued outside of the study remit.

For equity between treatment groups and to establish similar baseline data, all patients in each group were offered a similar pattern of eight treatments over four weeks.

### The measures

Whilst many potential tools are available for identifying psychosocial factors [34,35] and focus on various aspects of psychological distress [36-40] the Linton & Hallden questionnaire was chosen as it was devised to evaluate a broad spectrum of psychosocial factors linked to development of chronic non-specific low back pain [28,41]. It has proven 'test-retest' reliability [41] receives widespread support [42,26], and is quick and easy to administer in the clinical setting. Subjects were categorised by LH score into high (106 and above) and low (below 106) groups as a score of 105 out of a possible 210 has previously indicated a relatively high level of risk of developing long term pain and disability [43]. Linton & Hallden [41] found a significant relationship between score and prognosis such that a score of 106 and above had 86% sensitivity and 75% specificity for absenteeism from work.

The RMQ covers a variety of activities of daily living, is self-administered and easy to complete, has proven validity [44], excellent reliability [45] and superiority over other functional status questionnaires in terms of sensitivity to change [46]. The PRI and VAS components of the SF McGill were selected as they provide information regarding the multi- and uni-dimensional experience and intensity of pain respectively. The total PRI has good test-retest reliability (0.76) [47] and proven validity [48,49]. The VAS, was included as a second pain measure since it has been shown to be sensitive to interventions that alter the sensory experience of pain [50].

### Data Analysis

The data from the outcome measures were entered into SPSS for Windows software package (v.11) and subjected to descriptive and inferential analysis. Baseline data were compared between treatment groups for age, gender, employment status, chronicity, LH score, and the outcome measures of RMQ, PRI, VAS. The differences between post-treatment and baseline outcome measures for manual therapy and active rehabilitation were compared using an independent samples t test. However, to allow for possible confounding variables and investigate the possibility that the relative effects of manual therapy versus active rehabilitation differ according to LH score, each post-treatment outcome was regressed on treatment group, the corresponding baseline measure, gender, employment, LH (high or low), age, chronicity and the interaction between LH and type of treatment. A significance level of 0.05 was used for each test. Ethical approval was obtained.

## Results

57 patients were invited to participate in the study, 6 patients declined because of the commitment to attend twice a week. Of the 51 entering the study 39 completed (see Figure 1); 12 patients (8 from the active rehabilitation group and 4 from the manual therapy group) were lost through attrition despite follow up. (Only two were contactable: one had altered work circumstances and one had unforeseen family situations that made attendance impossible). An administrative oversight resulted in one additional patient being allocated to the High LH active rehabilitation group (n = 15) instead of the High LH manual therapy group (n = 13). Table 1 shows the demographic and baseline data and tests for differences between the active rehabilitation and manual therapy interventions for those completing the study. No significant differences were found between the two treatment groups at baseline.

**Table 1:** Demographic and baseline statistics by treatment group. AR = active rehabilitation; MT = manual therapy; NSLBP = non-specific low back pain; VAS = visual analogue scale; PRI = pain rated index; RMQ Roland Morris Questionnaire.

	**AR Group**	**MT Group**	**p**
Number	19	20	
Age (range, median, mean ± sd)	18–63, 42	29–66,48	0.401
	43.0 ± 13.3	46.4 ± 12.1	
Gender (male : female)	8 male : 11 female	12 male : 8 female	0.351
Employment status	11 employed	14 employed	0.396
	1 unemployed	0 unemployed	
	6 retired	3 retired	
	1 sick leave	3 sick leave	
Chronicity (weeks since onset of NSLBP)	7–312, 20	7–208, 24	0.355
Range, median, Mean ± sd	47.8 ± 71.4	41.8 ± 46.2	
Linton & Hallden Score	61–149, 106	60–150, 103.5	0.989
Range, median, mean ± sd	105.7 ± 23.7	106.3 ± 24.6	
High or low	9 low, 10 high	10 low, 10 high	
VAS (Range, median, Mean ± sd)	2–100, 42	2–70, 46.5	0.934
	41 ± 25.3	41.6 ± 18.9	
PRI (Range, median, Mean ± sd)	1–25, 11	2–38,15	0.235
	11.7 ± 6.3	15.1 ± 8.9	
RMQ (Range, median, Mean ± sd)	2–17, 9	2–21, 7	0.270
	9.2 ± 4.3	8.0 ± 5.3	

The changes between baseline and post-treatment appeared to be normally distributed for all three outcome measures. Further, Levene's test confirmed that equality of variance between groups could be assumed. For all outcome measures the mean change in manual therapy was greater than the mean change in active rehabilitation, mean difference for RMQ 3.6, 95% CI 1.1 – 6.2, p = 0.006, PRI 3.6, 95% CI 2.0 – 12.2, p = 0.007 and for VAS 15.0, 95% CI -1.1, 31.2, P = 0.067). These are reported in Table 2.

**Table 2:** Two sample comparison of change in treatment (pre – post) by treatment group. AR = active rehabilitation; MT = manual therapy; NSLBP = non-specific low back pain; VAS = visual analogue scale; PRI = pain rated index; RMQ Roland Morris Questionnaire.

	**Group**	**n =**	**Mean**	**Std dev.**	**Difference in means MT-AR (95% CI)**	**p**
VAS	AR	19	3.3	26.3	15.0 (-1.1,31.2)	.067
	MT	20	18.3	23.5		
RMQ	AR	19	0.6	3.2	3.6 (1.1,6.2)	.006
	MT	20	4.2	4.5		
PRI	AR	19	-0.1	7.2	7.1 (2.0,12.2)	.007
	MT	20	7.0	8.5		

Table 3 compares the mean difference between treatments (active rehabilitation versus manual therapy) for subjects with low and high LH scores. Those with high LH scores show a larger mean difference between treatments for RMQ and PRI, but a smaller one for VAS.

**Table 3:** Difference between treatment mean (AR – MT) for high and low LH scores. AR = active rehabilitation; MT = manual therapy; VAS = visual analogue scale; PRI = pain rated index; RMQ Roland Morris Questionnaire.

	LOW	HIGH
RMQ	0.556 – 3.300 = -**2.744**	0.600 – 5.100 = -**4.500**
PRI	2.889 – 5.900 = -**3.011**	-2.800 – 8.100 = -**10.900**
VAS	8.444 – 26.900 = **-18.456**	-1.400 – 9.700 = -**11.100**

Table 4 shows the results from regressions of post-treatment outcome on the corresponding baseline outcome, treatment group, demographic variables (age, gender, status of employment), chronic nature of condition, high or low LH score, and allowing a possible interaction between high or low LH score and treatment group.

**Table 4:** Regression of outcomes on demographic and baseline values, treatment group and LH high or low. AR = active rehabilitation; MT = manual therapy; VAS = visual analogue scale; PRI = pain rated index; RMQ Roland Morris Questionnaire; Unemp = unemployed.

	**VAS**		**PRI**		**RMQ**	
**Parameter**	**Coefficient (95% CI)**	**p**	**Coefficient (95% CI)**	**p**	**Coefficient(95% CI)**	**p**

Treatment (AR)	15.8 (-6.0,37.5)	0.149	7.9 (0.4,15.5)	0.041	3.9 (0.1,7.7)	0.043
Male	-13.8 (-29.8,2.2)	0.089	-5.9 (-11.6,-.13)	0.045	-3.9 (-6.7,-1.2)	0.007
Employed*	7.0	0.456	0.2	0.941	2.0	0.250
Off sick*	24.7	0.107	12.3	0.021	3.6	0.169
Unemp*	24.3	0.295	22.8	0.008	4.7	0.250
LH Low	-8.5 (-28.9,12.0)	0.405	4.3 (-2.8,11.3)	0.228	0.9 (-3.0,4.8)	0.637
Age	-0.91 (-0.6,0.6)	0.977	0.1 (-0.1,0.3)	0.425	0.004 (-0.113,0.120)	0.951
Chronicity	-0.03–0.2, 0.1	0.648	-0.01 (-0.05,0.04)	0.788	-0.008 (-0.032,0.017)	0.527
AR × LH low	-3.7 (-33.2,25.9)	0.802	-5.6 (-15.5,4.4)	0.259	-0.6 (-5.6,4.5)	0.820
Baseline	0.28	0.098	0.6 (0.3,1.0)	0.001	0.7 (0.4,1.1)	0.000

* compared to retired/not looking for work

RMQ showed a significant difference between groups (mean 3.9, 95% CI 0.1 – 7.7, p = 0.043). Gender (p = 0.007) was the only other variable with p < 0.10, male having an adjusted mean score 3.9 lower. Treatment effect was significant (mean 7.9, 95% CI 0.4 – 15.5, p = 0.041) for PRI. Employment (p = 0.011) and gender (p = 0.045) were both significant predictors of the change score. Treatment group was borderline significant (p = 0.055) for VAS, with final adjusted active rehabilitation mean score 15.8 higher than manual therapy (95% CI -6.0 to 37.5). Of the potential confounders, none were significant and only gender had p < 0.10.

The LH score was not significant for any outcome variable (p = 0.699 for RMQ, 0.611 for PRI, p = 0.405 for VAS). None of the interaction effects were significant (p = 0.820, 0.259, 0.802 for RMQ, PRI and VAS respectively). However, confidence intervals in both cases were wide (Table 4) so this does not preclude the existence of a non-zero main effect or interaction.

## Discussion

This pilot study found no patients were referred with onset of non-specific low back pain of 6 weeks duration, and only 2 people who completed the study had suffered less than 8 weeks. This supports previous research that it is difficult to implement CSAG recommendations within the specific time frame (and therefore effectively research the outcomes of any implementation) due to the delay in patient presentation [42,51]. It is also a consequence of recruitment being at the point of referral to physiotherapy as previous research suggests general practitioners have a lack of awareness of the guidelines and the need for early referral [52,53].

The age and gender range of subjects recruited to the study appear to reflect the clinical population normally referred to the host physiotherapy department, although the attrition rate was higher than normally recorded. The specific process of allocation by LH score, whilst essential to balance numbers of low and high scores within each treatment arm, is in direct contrast to the normal practice of negotiated management and mutually formulated treatment goals and this may have generated higher attrition. It may also be the case that recruitment via the contact physiotherapist, rather than via other sources of referral, may have introduced a selection bias towards attrition at the point of obtaining consent and this would warrant further consideration in further studies. For future studies, access to patients with acute non-specific low back pain may need to be through direct access to minimise time delays in presentation and to reduce 'gate-keeper' influences – for example, in the initial selection of patients for physiotherapy and again at the point of attendance – in recruitment.

23.5% (n = 12) of the 51 subjects recruited did not complete, with 8 subjects from the active rehabilitation group (5 from the High LH active rehabilitation group) lost prior to completion. Post-hoc analysis of the subjects lost to the trial (n = 12) compared to those subjects who completed the study (n = 39) found that younger, unemployed people with higher psychosocial risk scores tended to 'drop out' of treatment. The mean age of those lost to the study was 10 years younger than those who completed the study; mean LH score was 116 as opposed to 106 in the 'study' group. All subjects 'lost' were either 'unemployed' or on sick leave from work, as opposed to only 5 of the 39 subjects who completed the study. Such attrition is not unusual in trials studying non-specific low back pain: Geisser *et al*, [15] lost 28% of subjects during their trial period, and Frost *et al*, [10] lost 30%, although most were lost to follow up. Goldby *et al*, [11] recruited 346 subjects of which 302 entered the study but 22% withdrew or were withdrawn during the intervention period, and 50% were lost to the trial by the end of the two-year study period. In terms of the higher attrition in the active rehabilitation group, Lewis *et al*, [12] reflected similar findings although of the 18 subjects (from 80) lost during their study period only 7 (17.5%) were from the active exercise group (n = 40). Whilst there is a lack of information regarding population characteristics of those who fail to complete trials relating to non-specific low back pain, we note our population reflects key findings by Hay *et al*, [30] that young male unemployed subjects are more likely to fail to complete treatment. We are also mindful that a patient's positive attitudes to treatment may have an influential effect on their perceived benefits and compliance [18] and the potential for a greater number of patients in this sample (through their presentation and possible request for referral to physiotherapy) to hold more strongly with beliefs related hands-on practice and intervention. This was possibly indicated at the end of the trial period when patients for whom treatment had not satisfactorily resolved their clinical signs and symptoms were offered the opportunity to undertake alternative treatments: 4 patients in the active rehabilitation group requested manual therapy in contrast with no members of the manual therapy group requesting active rehabilitation. However, no baseline data was collected about patient preference and so this potential influence is highlighted for further study.

In final consideration of the effects of attrition, it is noted that no intention to treat analysis was carried out. Thus, whilst table 1 and the associated population analysis demonstrates sound randomisation at recruitment, it cannot be assumed that the population completing the trial had not been subject to further bias linked to the reasons for attrition. As noted above, only two subjects were contactable following their withdrawal from the trial and so further outcome data was unavailable.

Outside of the noted problems of failure to recruit acute patients, potential selection bias and attrition, the pilot design was considered as feasible for further studies.

In relation to the second aim of the study, to provisionally consider the data generated we note that subjects following manual therapy intervention demonstrated greater improvement in all outcome measures and our results favoured this first line intervention for this sample population. We are mindful of the small sample size and the potential effect of this on any assumed treatment effect demonstrated within the data but offer the preliminary analysis and discussion with respect to this.

The trend within our results is to support previous findings for manual therapy [13,14,16] but also to add conflict to studies that have found in favour of active rehabilitation [54] or stabilisation exercises [11,12]. However, specifically, as we chose to replicate normal practice, our trial could not incorporate a 'no intervention group' control and we can offer no conclusion as to whether either manual therapy or active rehabilitation was more beneficial than 'normal' recovery; a feature of pragmatic clinical trials.

Further caution is applied to our results in that we have considered very short-term effects (immediate post-treatment) associated with manual therapy and it is recognised that follow-up would be a requirement of any trial. Previous research considering the longer-term benefit of various treatment approaches has been largely inconclusive [55]. For example, Aure *et al*, [13] found manual therapy demonstrated significantly greater improvements than active rehabilitation in short and long term follow up, and the UK BEAM trial [16-19] identified significant improvements for manipulation and exercise 12 months post-intervention. This is in direct contrast to other studies [30,54,56] that found no evidence that manual therapy was superior to other conventional treatments or exercise over time.

In contrast to CSAG recommendations [2] that active rehabilitation should be used for those patients with non-specific low back pain who are not improving at 6 weeks, we identify a trend that suggests manual therapy may still be effective in sub-acute and chronic populations, and that this may be more effective than active rehabilitation. We are mindful that the recruitment excluded patients who had undergone physiotherapy within the past three months and as such our population does not necessarily represent a population for whom previous manual therapy has proven unsuccessful, which is the intention of the CSAG guidelines. Active rehabilitation may have a greater influence on those clients who have not responded to previous treatment [57], which our design did not accommodate. Again, given the small numbers included in this pilot and the means of recruitment, it may also be that our study population did not sufficiently recruit patients who are receptive to a cognitive approach [41,58]. It may also be the case that the small numbers lead to insufficient randomisation of characteristics associated with influence on treatment outcomes of positive or negative beliefs related to passive or active treatment approaches; patients have previously perceived manual therapy to be more passive, but more associated with hands-on expertise, than active exercise [18].

Our design sought to specifically consider the influence of psychosocial factors as a potential influence on the effectiveness of treatment mode, as suggested by CSAG guidelines [2]. From our preliminary results, psychosocial factors, as defined and measured through the LH Questionnaire, did not significantly influence outcome measures and provides no support for profiling patient subgroups based on psychosocial screening. For RMQ and PRI smaller difference in mean scores were found in the 'low' LH group. For VAS, however, a bigger mean difference occurred in the 'low' group. This lack of demonstrable significant influence may be related to the late onset of treatment [31] or the higher attrition rate in the group in the absence of an intention to treat analysis.

Retrospective power calculations should be interpreted with caution. However, using the estimated error variances of the change in outcomes and considering a balanced design with two levels of LH score and two treatment groups, a sample size of (4 × 9 =) 36 is sufficient to detect a difference in therapy means between LH groups of 52.9, 8.6 and 16.7 for VAS, RMQ and PRI respectively with 80% power. This study was, therefore, only sufficiently powered to detect very large mean differences between active rehabilitation and manual therapy for low and high LH scores. None-the-less, the data suggest that there is scope for more research in this area with a greater sample size to explore this possible interaction further.

Given that this is a pilot study we have not considered the issues of multiple comparisons because the main aim of the study requires the three statistical tests comparing manual therapy and active rehabilitation and the corresponding tests of interaction only. This would need address in the main study.

## Conclusion

This was a small feasibility study and the data should be interpreted with great caution. All subjects with non-specific low back pain undertaking manual therapy as first line intervention fared significantly better in reduced pain intensity and increased functional outcomes than those following an active rehabilitation treatment programme over a 4-week period. There was no evidence that this effect differed between subjects with 'high' or 'low' LH scores.

The preliminary analysis of the data was to consider whether this pilot study could support wider enquiry, via a pragmatic randomised trial, into the comparative benefits of active rehabilitation versus manual therapy or a combination of both approaches at different stages of non-specific low back pain history. We believe the design and outcome measures selected are appropriate, and the results sufficiently interesting enough to warrant further study with the following recognition and recommendations:

1) The study was powered to detect only major interaction effects and the small sample size recruited indicates a priori power analysis should be performed to determine minimum sample size required

2) The potential placebo influence where different grades of physiotherapist managed the different treatment arms is consistent with the 'real life' clinical environment.

3) The main study may be best served by a pragmatic study design. A randomisation process with stratification based on the LH score may be employed to subgroup rather than purposive allocation based on the LH score.

4) Recruitment via direct access and/or specific recruitment strategies to address the delay in presentation to physiotherapy and the potential selection bias that exists at each contact point for patients (e.g., general practitioner, physiotherapist), and addresses patient self-selection should be considered.

5) Evidence of longer-term effect was not explored and the results are confined to the immediate post-treatment period. We suggest that the main study should consider long term follow up of the patients' outcome including investigation of the population lost to treatment.

6) Possible bias was introduced by using treating physiotherapist to administer the final questionnaires to patients to complete. Although the patient returned them separately in a sealed brown envelope (therapist was blind to the actual results) it is recommended that a research assistant administer all questionnaires.

## Competing interests

The author(s) declare that they have no competing interests.

## Authors' contributions

EH conceived of the initial project, study design, acquisition of data, initial analysis and write-up as part of MSc. EH drafted the current manuscript. RS acted as supervisor throughout the MSc process and was involved in critically revising the subsequent document in readiness for submission to publication.

LS performed statistical analyses of original MSc data to prepare current document and gave valuable input to prepare for submission to publication. All authors approve this final submission.

## Pre-publication history

The pre-publication history for this paper can be accessed here:


